# ‘When you give kindness out, you get it back ten times more’: Ontario adults’ prosocial behaviour during the first 16 months of the COVID-19 pandemic

**DOI:** 10.1371/journal.pone.0288720

**Published:** 2023-07-17

**Authors:** Katie J. Shillington, Julia Yates, Leigh M. Vanderloo, Shauna M. Burke, Victor Ng, Patricia Tucker, Jennifer D. Irwin

**Affiliations:** 1 Faculty of Health Sciences, Health and Rehabilitation Sciences Program, University of Western Ontario, London, Ontario, Canada; 2 Faculty of Health Sciences, School of Occupational Therapy, University of Western Ontario, London, Ontario, Canada; 3 Faculty of Health Sciences, School of Health Studies, University of Western Ontario, London, Ontario, Canada; 4 Children’s Health Research Institute, Lawson Health Research Institute, London, Ontario, Canada; 5 Schulich School of Medicine and Dentistry, University of Western Ontario, London, Ontario, Canada; 6 Department of Family and Community Medicine, University of Toronto, Toronto, Ontario, Canada; 7 Division of Professional and Practice Support, College of Family Physicians of Canada, Mississauga, Ontario, Canada; Aravind Eye Hospital and Post Graduate Institute of Ophthalmology, INDIA

## Abstract

The COVID-19 pandemic has provided a collective opportunity to engage in prosocial behaviours, including kindness; however, little is known about the long-term impacts of the pandemic on such behaviours. As a part of a larger study (Health Outcomes for Adults During and Following the COVID-19 Pandemic), the purpose of this mixed methods research was two-fold: (1) to quantitatively explore adults’ prosocial behaviour over time during the first 16 months of the pandemic in Ontario, Canada (April 2020-August 2021); and, (2) to more deeply explore, via focus groups, a sub-sample of Ontario adults’ lived experiences of prosocial behaviour (assessed March 2022). A total of 2,188 participants were included in this study, with the majority of participants identifying as female (89.5%). At three time points, participants completed online questionnaires which included demographics, Prosocialness Scale for Adults, and three global kindness questions. A subset of participants (*n* = 42) also participated in one of six focus groups exploring their experiences of prosocial behaviour during the pandemic. A series of one-way repeated measures ANOVAs revealed that participants’ self-reported prosocial behaviour increased significantly over time, while participants’ awareness of kindness, engagement in acts of kindness, and view of kindness as crucial significantly decreased. Thematic analysis revealed three main themes: (1) shift in prosocial behaviour during the pandemic; (2) kindness from various perspectives; and, (3) prosocial burnout. This study provides insight into the longer-term effects of the pandemic on adults’ prosocial behaviours and should be leveraged to help understand how individuals respond in times of crises.

## Introduction

The ongoing COVID-19 pandemic has necessitated public health protections globally [e.g., 1,2]. Inclusive of physical distancing, mask-wearing, and vaccinations, these protections have required behaviours that largely hinge upon community adherence [3,4]. Adherence to public health guidelines has been recognized as a form of prosocial behaviour [5], which is defined as “voluntary behaviour intended to benefit another, such as helping, donating, sharing, and comforting” [6, pp1668] and can include many domains (i.e., compassion, caring, love, sympathy, empathy, altruism, and kindness; [7,8]).

While the COVID-19 pandemic is associated with much disruption and trauma for adults globally [9], it has simultaneously provided a unique opportunity for people to engage in prosocial behaviour [5]. Pre-pandemic, Vollhardt [10] asserted that experiencing adverse events is associated with an increase in prosocial and helping behaviour. As such, it is possible that the ongoing COVID-19 pandemic has triggered motivation to engage in prosocialness. Prosocial behaviour has been associated with positive wellbeing [11–13] and given the difficulties experienced during the COVID-19 pandemic, prosocial behaviours appear to contribute positively to wellbeing [14–16]. For example, Varma and colleagues [15] investigated the effectiveness of prosocial behaviour as a strategy to promote wellbeing during the COVID-19 pandemic. The authors conducted two studies with 1,623 adults (M_age_ = 26) who were randomly assigned to engage in other- or self-beneficial behaviours [15]. It was found that, compared to non-prosocial or self-beneficial action, engagement in prosocial behaviour led to significantly increased positive affect, empathy, and social connectedness [15]. Similarly, Datu and colleagues [17] investigated the impact of gratitude and kindness interventions on the positive emotions (e.g., happy, joyful) of undergraduate students (*N* = 107) during the pandemic. Participants in the study were randomly assigned to one of three conditions: (1) kindness; (2) gratitude (which is “a felt sense of wonder, thankfulness, and appreciation for benefits received” [18, pp327]); or (3) control. The authors found that individuals assigned to the gratitude and kindness conditions scored significantly higher on positive emotion measures in comparison to those in the control group [17]. Moreover, Raposa and colleagues [19] showed that prosocial behaviour can positively influence one’s neurological system. Specifically, when an individual engages in a kind act, oxytocin—a hormone that helps to mitigate feelings of fear and stress—is released [19]. As such, prosocial behaviour may be considered an effective coping strategy for adults experiencing distress during the pandemic [19].

In addition to the psychological benefits associated with kindness, as described above, engagement in prosocial behaviour has been linked to social belonging [16]. Social belonging has been described as “a sense of deep connectedness, affiliation, and integration with a social group or community” [9, pp2] and is crucial for wellbeing [20–22]. During the COVID-19 pandemic, social connection has been limited due to necessary public health protections, such as lockdowns, isolation, and physical distancing practices [23]. It has been found that some of these protections have contributed to feelings of social isolation and loneliness [24], which can be exacerbated by additional pandemic-related stressors (e.g., social, financial, health; [16]). Given that engagement in prosocial behaviour can increase feelings of social connectedness [16], it may also serve as a buffer against pandemic-related loneliness and social isolation. In fact, researchers surveyed 437 undergraduate students in the United States during the early stages of the pandemic (April 2020) and found that being on the receiving end of prosocial behaviour was associated with statistically significantly greater perceptions of belongingness [14].

There have been large variations in pandemic experiences globally and provincially in Canada (e.g., length and degree of public health protections enforced, social isolation, loneliness, decreased wellbeing). Gaining insight into adults’ prosocial behaviour, inclusive of kindness, as well as their lived experiences may be beneficial in creating supports specific to the needs of Ontario adults. Notably, kindness was selected as a topic in the research because of the previously established relationship between the construct of kindness and several positive mental health outcomes such as increased happiness [25–27], resilience [28], and positive mental health and wellbeing [29–32]. Understanding how the pandemic timeframe would impact prosocial behaviour and its construct of kindness was particularly important given (1) the established relationship between kindness and mental health and (2) the uncertainty surrounding how the pandemic might impact various correlates of adults’ mental health, including kindness. To this end, and as a part of a larger study titled *Health Outcomes for adults during and following the COVID-19 PandEmic (HOPE)*, the purpose of this paper was two-fold: (1) to quantitatively assess adults’ prosocial behaviour—including kindness—over time during the first 16 months of the pandemic in Ontario, Canada (April 2020-August 2021); and, (2) to more deeply explore, via focus groups, a sub-sample of Ontario adults’ lived experiences of prosocial behaviour (assessed March 2022).

## Methods

### Study design

*HOPE* is an ongoing, longitudinal study that explores adults’ lifestyle-related health behaviours and outcomes, including physical activity, sedentary behaviour, sleep, diet, mental health, wellbeing, and prosocial behaviour, during and following the COVID-19 pandemic in Ontario, Canada [33–35]. As indicated above, the current paper reports on the prosocial data quantitatively measured via survey and qualitatively explored via focus groups. The methods (i.e., study design, study procedures, recruitment, measures, data analysis) for this research have been detailed elsewhere [33–35].

### Study procedures

Participants were primarily recruited for *HOPE* via social media platforms. To be eligible for the study, participants were required to be: (1) an Ontario resident; (2) between the ages of 30–59 years at baseline; and (3) able to read and write in English. *HOPE* included three time points: (1) time point 1 (T1; April 24-July 13, 2020); (2) time point 2 (T2; July 29-August 30, 2020); and, (3) time point 3 (T3; July 29-August 30, 2021). When interested participants clicked the online study advertisement, they were directed to a survey that included the letter of information, eligibility, consent process, and the T1 questionnaires. Upon reading the letter of information and confirming their eligibility via self-declaration, participants were asked to provide electronic consent by selecting/clicking on “I consent to begin this study” in the online survey. Individuals who consented to participate were then directed to the T1 questionnaires. Individuals who selected “I do not consent, I do not wish to participate” were redirected to the end of the survey. The same questionnaires were administered at T2 and T3, with the exception of some participant demographics.

At T3, participants were invited to participate in a focus group. Those who expressed interest were provided with the letter of information, asked to confirm their eligibility, provide consent, submit their participant ID, and select their availability for a focus group date and time. Per the guidance of Hennink and colleagues [36] regarding the number required to reach theoretical saturation, six focus groups occurred March 6–12, 2022. The focus groups took place via Zoom with a moderator (KS), assistant moderator (JY), and three note-takers (JC, KF, ZR) and ranged from 60–90 minutes in length. Theoretical saturation was reached by the fourth focus group and confirmed by the sixth focus group. To diminish social desirability bias [37], participants were told there were no right or wrong answers at the beginning of each focus group. To support the credibility of the data, the moderator member-checked between questions and summarized responses at the end of each focus group to confirm that the responses were accurate from participants’ perspectives (per Guba & Lincoln, [38]). Focus groups were audio-recorded and transcribed verbatim by Zoom and checked for accuracy by a member of the research team.

### COVID-19 context at the time of data collection

Data collection at T1 and T2 occurred during the first wave of the pandemic, wherein Ontario was in a lockdown (e.g., closure of schools, businesses, and non-essential services) and case counts were at a peak [39]. One year follow-up data collection (T3) occurred from July to August 2021, during which the province had re-opened, meaning indoor services with larger numbers of people could resume [40]. Masks and vaccinations were enforced; however, despite the protection efforts, case counts increased at the end of August, signalling a fourth wave [39]. As described, the focus groups occurred in March 2022; at this time public health measures lifted which resulted in an increase in COVID-19 transmission, as well as hospital and intensive care unit occupancy, signaling a sixth wave of the pandemic [41].

### Tools

#### Quantitative

At T1 demographic questions assessed participants’ age, sex, gender, ethnicity, geographic location, employment status, income, educational attainment, marital status, COVID-19 diagnosis, and presence of mental health conditions. The T2 and T3 demographic questionnaires included questions pertaining to the extent to which participants’ incomes may have changed over the pandemic, employment status, COVID-19 diagnosis (at any time point), and presence of mental health conditions.

A modified 16-item Prosocialness Scale for Adults (PSA; [42]) was utilized to measure participants’ prosocial behaviour on a 5-point Likert scale ranging from 1 (never/almost never) to 5 (always/almost always). Tool items reflected prosocial actions including sharing, helping, taking care of others, and feeling empathetic [42]. The tool was slightly altered from its original form for use in *HOPE* to be more conducive to public health recommendations at the time of administration—six questions were removed and two questions were re-worded (see [35] for question details). As such, the revised tool included eight original and two modified items. The modified PSA had a high level of internal consistency, as determined by a Cronbach’s alpha of 0.89.

Given that the PSA did not measure kindness specifically, nor in the context of the COVID-19 pandemic, three kindness questions were added. The three questions were created by the research team to measure participants’ understanding and experiences of kindness during the pandemic, using the same 5-point Likert scale described above. Specifically, participants were asked about the extent to which they: (1) were aware of kindness around them during the COVID-19 pandemic; (2) purposefully engaged in deliberate acts of kindness during the pandemic; and (3) viewed kindness as a crucial component of their COVID-19 pandemic experience.

#### Qualitative

A semi-structured interview guide was followed, with questions pertaining to the prosocial behaviour of Ontario adults, including preliminary quantitative findings (see S1 Appendix). Quantitative findings were used to inform the focus group guide, such that participants were asked to share their insights about the study findings, specifically regarding what resonated or did not resonate with them.

### Data analysis

#### Quantitative

To determine whether there was a statistically significant difference in participants’ self-report prosocial behaviour and kindness during the COVID-19 pandemic (from T1 to T3), a series of one-way repeated measures ANOVAs were conducted. A Bonferroni correction was applied to account for multiple comparison bias in post-hoc analyses, and multiple imputation was used to handle missing data. All data analyses were completed in SPSS (version 28.0.1.1).

#### Qualitative

Focus group transcripts were organized using Quirkos qualitative analysis software [43] and an inductive content analysis approach [44] was utilized following the method for thematic analysis outlined by Braun and Clarke [45]. Two researchers (KS, JY) independently and simultaneously familiarized themselves with the data by reading and re-reading the transcripts while making notes [45]. Next, the researchers generated initial codes by analyzing the entire dataset and then collating the codes into potential themes [45]. The two researchers then met to define and name the themes to create a tentative codebook [45]. To ensure codebook accuracy, five researchers (KS, JY, JC, KF, ZR) were split in dyads and each dyad was assigned one transcript to code using the preliminary codebook, making note of themes/definitions that needed refinement. The use of multiple coders was to support confirmability (per Guba & Lincoln, [38]). Prior to meeting with the larger group, each dyad met individually to discuss the coding structure, following their individual review of the transcripts. The five researchers then met to review the codebook themes to ensure they related to the coded extracts (step 1) and then to the entire dataset (step 2); refinements were made to themes and definitions as necessary [45]. Using the revised codebook, researchers were again split into dyads and each dyad coded two transcripts. Once analysis was complete, all Quirkos files were merged and exported. To support transferability, the methods and study procedures were documented to enable researchers to replicate the study (per Guba & Lincoln, [38]).

To help elucidate the popularity of specific examples of prosocial behaviours in which participants engaged, an additional analysis was deemed suitable and was therefore conducted within one of the themes. Specifically, a summative content analysis (per Hsieh & Shannon [46]) was conducted independently and simultaneously by two researchers (KS, JY). To determine the frequency of prosocial behaviours in which participants engaged. Initially, the researchers reviewed the transcripts and noted the frequency of prosocial behaviours mentioned by participants. Behaviours were then categorized into common groupings and assigned working titles. Upon finalizing the themes independently, the researchers met to agree upon final themes and count the number of examples of prosocial behaviours in each theme.

## Results

### Quantitative

#### Demographics

A total of 2,188 (*M*_age_ = 43.15; *SD* = 8.82) Ontario adults completed the survey. The majority identified as female (*n* = 1,743; 89.55%) and Caucasian (*n* = 1,789; 91.55%). The socioeconomic status of the sample was generally high, with the majority reporting full-time employment (*n* = 1,162; 59.22%), an annual income over $111,000 (*n* = 845; 43.07%), and college-level education or higher (*n* = 1,741; 88.69%). For full demographic details, refer to Table 1.

**Table 1:** Demographic information of survey participants.

Participant Characteristics (*N* = 2,188)	*n*	%
Age (years), *M (SD)*		
Total	43.15 (8.82)	
Sex		
Female	1,749	89.55
Male	200	10.24
I prefer not to answer	3	0.15
Not listed	1	0.051
Gender		
Female	1,743	89.57
Male	198	10.17
Non-Binary	2	0.10
I prefer not to answer	3	0.15
Ethnicity		
Arab	4	0.20
Black	9	0.46
Caucasian (White)/European	1,789	91.55
Chinese	22	1.12
Filipino	5	0.25
Indigenous	20	1.02
Japanese	4	0.20
Korean	3	0.15
Latin American	14	0.72
Maltese	1	0.051
Metis	3	0.15
South Asian	38	1.94
Southeast Asian	5	0.25
West Asian	2	0.10
West Indian	1	0.051
Multiracial	19	0.97
I prefer not to answer	14	0.72
Not listed	1	0.051
Employment Status at T1		
Employed full-time	1,162	59.22
Employed part-time	156	7.95
Casual	33	1.68
Unemployed	204	10.40
I prefer not to answer	10	0.51
Other	397	20.23
Employment Status at T2		
Employed full-time	503	58.15
Employed part-time	82	9.50
Casual	15	1.73
Unemployed	55	6.36
I prefer not to answer	6	0.69
Other	204	23.58
Employment Status at T3		
Employed full-time	489	62.61
Employed part-time	69	8.83
Casual	16	2.05
Unemployed	39	4.99
I prefer not to answer	5	0.64
Other	163	20.87
Income (T1)		
< $30,000	98	4.99
$30,000-$59,000	236	12.03
$60,000-$79,999	225	11.47
$80,000-$110,999	375	19.11
$111,000-$150,000	390	19.88
> $150,000	455	23.19
I prefer not to answer	183	9.33
Extent That Income Changed Since T1 (T2)		
Reduced	122	14.10
Stayed the same	678	78.38
Increased	65	7.51
Extent That Income Changed Since T1 (T3)		
Reduced	148	18.95
Stayed the same	428	54.80
Increased	205	26.25
Tested Positive for COVID-19 (T1)		
Yes	32	1.63
No	1928	98.37
Tested Positive for COVID-19 (T2)		
Yes	16	1.86
No	844	98.14
Tested Positive for COVID-19 (T3)		
Yes	25	3.20
No	756	96.80
Marital Status		
Single	242	12.33
Married/common law/engaged	1,535	78.20
Divorced/separated	156	7.95
Widowed	18	0.92
I prefer not to answer	12	0.61
Highest Level of Education		
Less than high school	24	1.22
High school	150	7.64
Community college/journeyman apprenticeship	618	31.48
University undergraduate degree	550	28.02
University graduate or degree or higher	573	29.19
I prefer not to answer	6	0.30
Other	42	2.14

*Note*. Time point 1 (T1) occurred from April-July 2020, time point 2 (T2) occurred from July-August 2020; and time point 3 (T3) occurred from July-August 2021. The total sample size was 2,188 participants; not all categories summed to equal the total sample due to missing data. Age was collected as a continuous variable.

Focus group participants (*n* = 42) were, on average, 42.74 years old (*SD* = 8.48), with most identifying as female (*n* = 30; 85.7%). The majority were Caucasian (*n* = 32; 91.4%) and the geographic location with the largest participant representation was London (*n* = 8; 22.86%). Most focus group participants reported full-time employment (*n* = 18; 51.43%), an annual income of $111,000 or above (*n* = 17; 48.58%), and an undergraduate-level education or higher (*n* = 23; 65.71%). For a detailed description of focus group participants, refer to Table 2.

**Table 2:** Demographic information of focus group participants.

Participant Characteristics (*n* = 42)	*n*	%
Age, *M (SD)*		
Total	42.74 (8.48)	
Sex		
Female	31	88.57
Male	4	11.43
Gender		
Female	30	85.71
Male	4	11.43
Non-Binary	1	2.86
Ethnicity		
Caucasian (White)/European	32	91.43
Indigenous	1	2.86
Multiracial	2	5.71
Employment Status		
Employed full-time	18	51.43
Employed part-time	2	5.71
Casual	1	2.86
Unemployed	4	11.43
Other	10	28.57
Income		
< $30,000	4	11.43
$30,000-$59,000	5	14.29
$60,000-$79,999	1	2.86
$80,000-$110,999	8	22.86
$111,000-$150,000	12	34.29
> $150,000	5	14.29
Highest Level of Education		
Less than high school	1	2.86
Community college/journeyman apprenticeship	10	28.57
University undergraduate degree	7	20.00
University graduate or degree or higher	16	45.71
Other	1	2.86
Marital Status		
Single	5	14.29
Married/common law/engaged	28	80.00
Divorced/separated	2	5.71
Tested Positive for COVID-19		
Yes	3	8.57
No	32	91.43

*Note*. The total focus group sample size was 42 participants; not all categories summed to equal the total sample due to missing data. Age was collected as a continuous variable.

#### Prosocial behaviour

**Fig 1:**
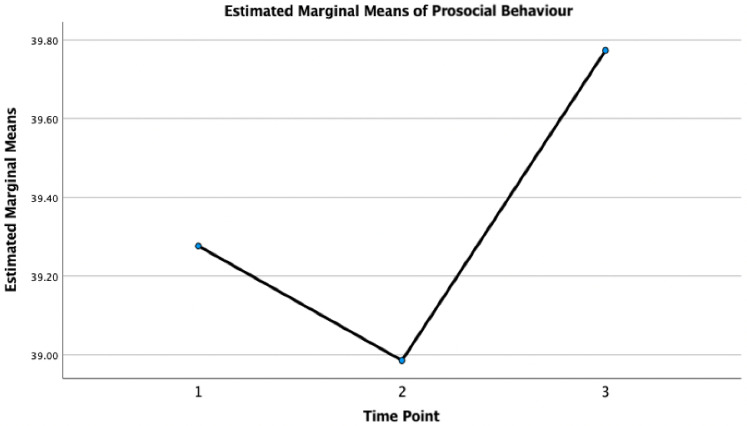
Participants’ prosocial behaviour over time. *Note*. Time refers to the three time points (i.e., 1 = time point 1 [April 24-July 13, 2020]; 2 = time point 2 [July 29-August 30, 2020]; and 3 = time point 3 [July 29-August 30, 2021].

A one-way repeated measures ANOVA revealed that there was a statistically significant difference in participants’ prosocial behaviour over time (see Fig 1). Post hoc testing revealed that participants’ prosocial scores decreased significantly from T1-T2 (M_difference_ = 0.29, *p* = 0.003, 95% CI = 0.079, 0.50), and increased from T1-T3 (M_difference_ = -0.50, *p* = < 0.001, 95% CI = -0.73 to -0.26) and T2-T3 (M_difference_ = -0.79, *p* = < 0.001, 95% CI = -1.00 to -0.58). The mean, standard deviation, and the *F*-ratio of the one-way repeated measures ANOVA for the PSA can be found in Table 3.

**Table 3:** The prosocial behaviour and kindness of participants over time.

Scale	Time Point 1 *M (SD)*	Time Point 2 *M (SD)*	Time Point 3 *M (SD)*	*F*-ratio
***Prosocialness Scale for Adults***				
Total Score (out of 50)	39.28 (5.65)	38.98 (5.77)	39.77 (5.62)	*F*(1.96, 4296.46) = 38.3, *p* = < 0.001*, η2p = 0.017
***Global Kindness Questions***				
I am aware of kindness around me during COVID-19	3.92 (0.79)	3.65 (0.82)	3.71 (0.89)	*F*(1.95, 4261.26) = 111.46, *p* = < 0.001*, η2p = 0.048
I purposefully engage in deliberate acts of kindness during COVID-19	3.51 (0.91)	3.41 (0.90)	3.40 (0.95)	*F*(1.95, 4263.12) = 18.34, *p* = < 0.001*, η2p = 0.008
I view kindness as a crucial component of my COVID-19 experience	3.87 (0.96)	3.69 (1.12)	3.72 (1.04)	*F*(2.00, 4371.32) = 32.44, *p* = < 0.001*, η2p = 0.015

*Note*. An asterisk (*) indicates statistical significance (*p* = < 0.05).

#### Kindness

**Fig 2:**
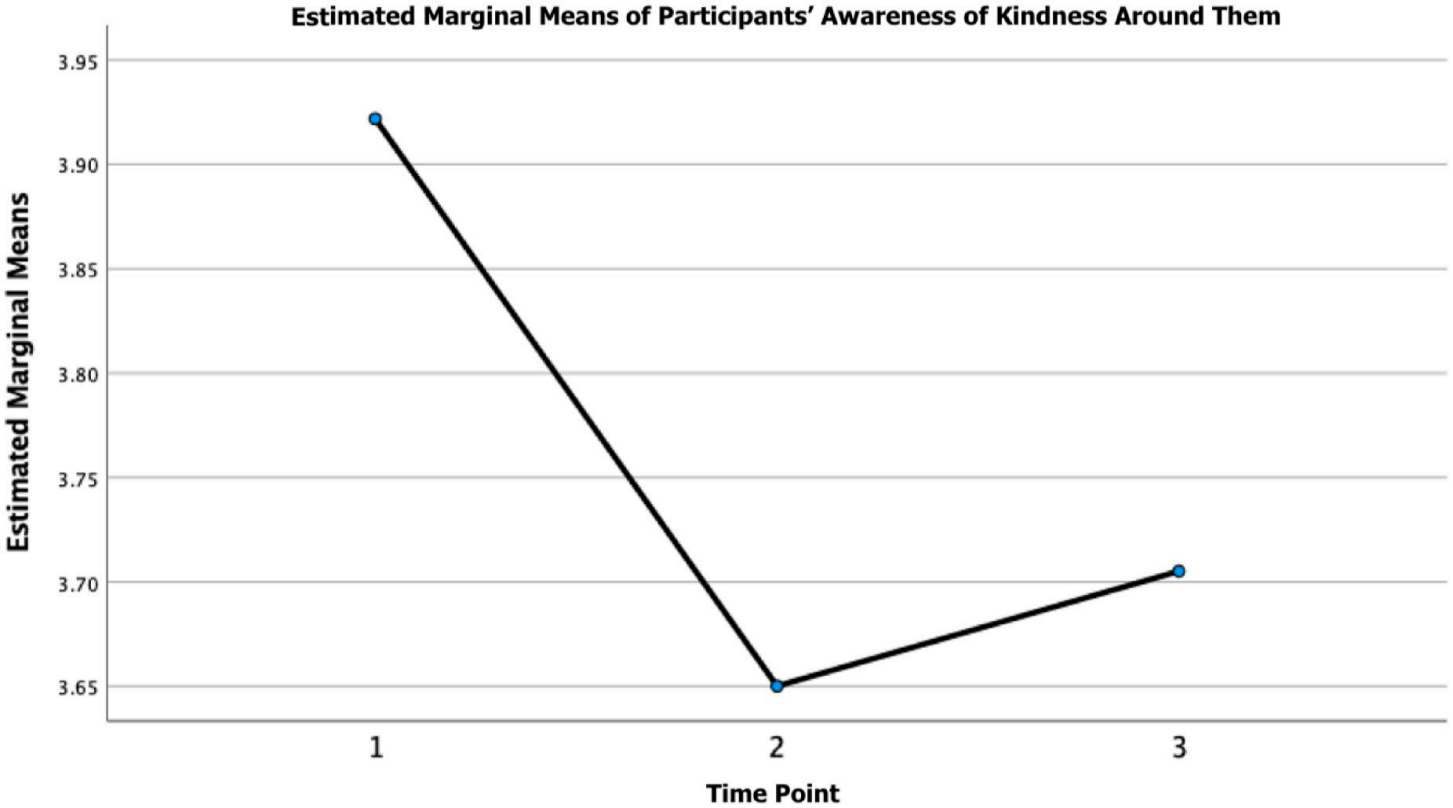
Participants’ awareness of kindness around them over time. *Note*. Time refers to the three time points (i.e., 1 = time point 1 [April 24-July 13, 2020]; 2 = time point 2 [July 29-August 30, 2020]; and 3 = time point 3 [July 29-August 30, 2021].

**Fig 3:**
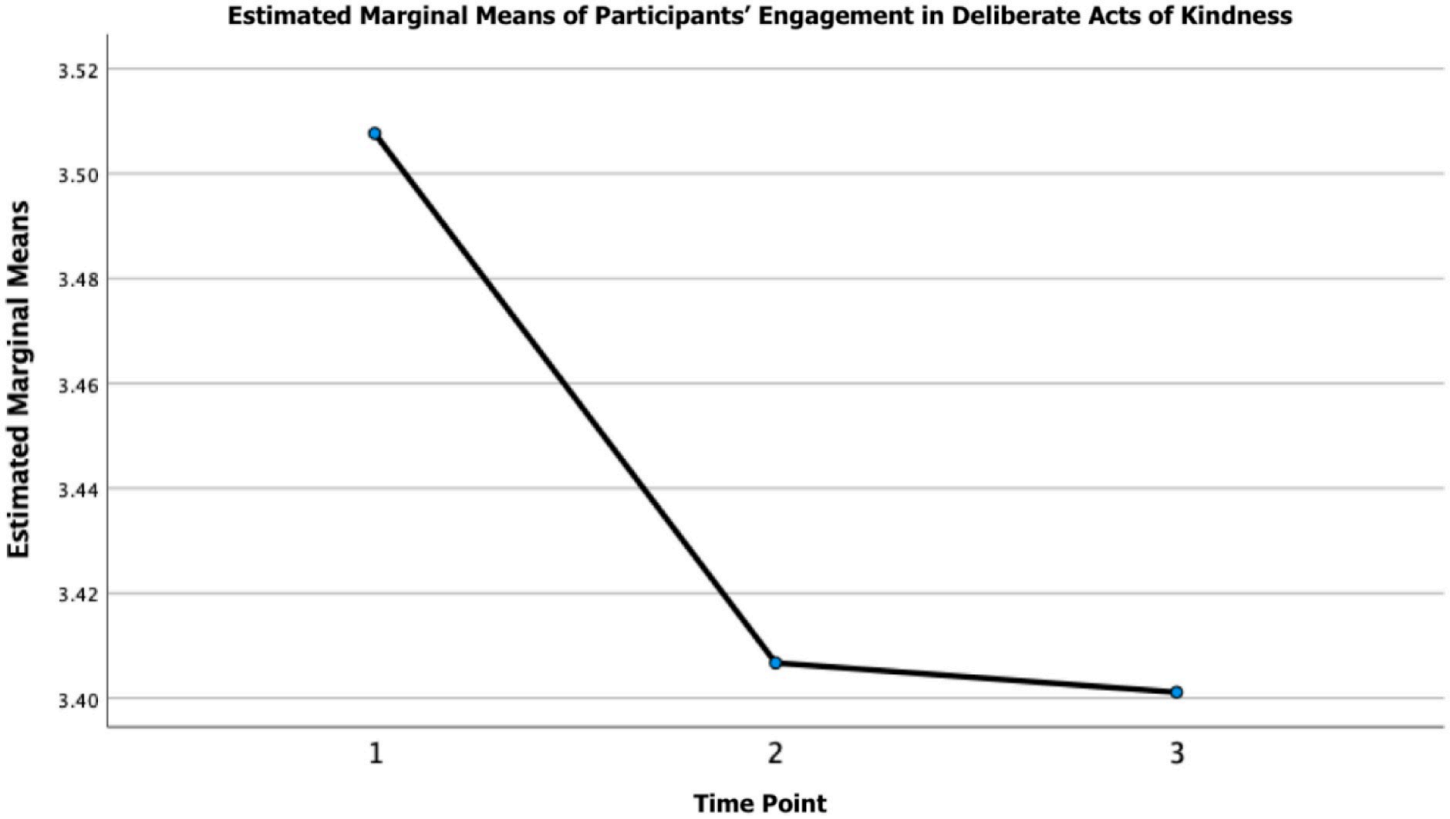
Participants’ engagement in deliberate acts of kindness over time. *Note*. Time refers to the three time points (i.e., 1 = time point 1 [April 24-July 13, 2020]; 2 = time point 2 [July 29-August 30, 2020]; and 3 = time point 3 [July 29-August 30, 2021].

**Fig 4:**
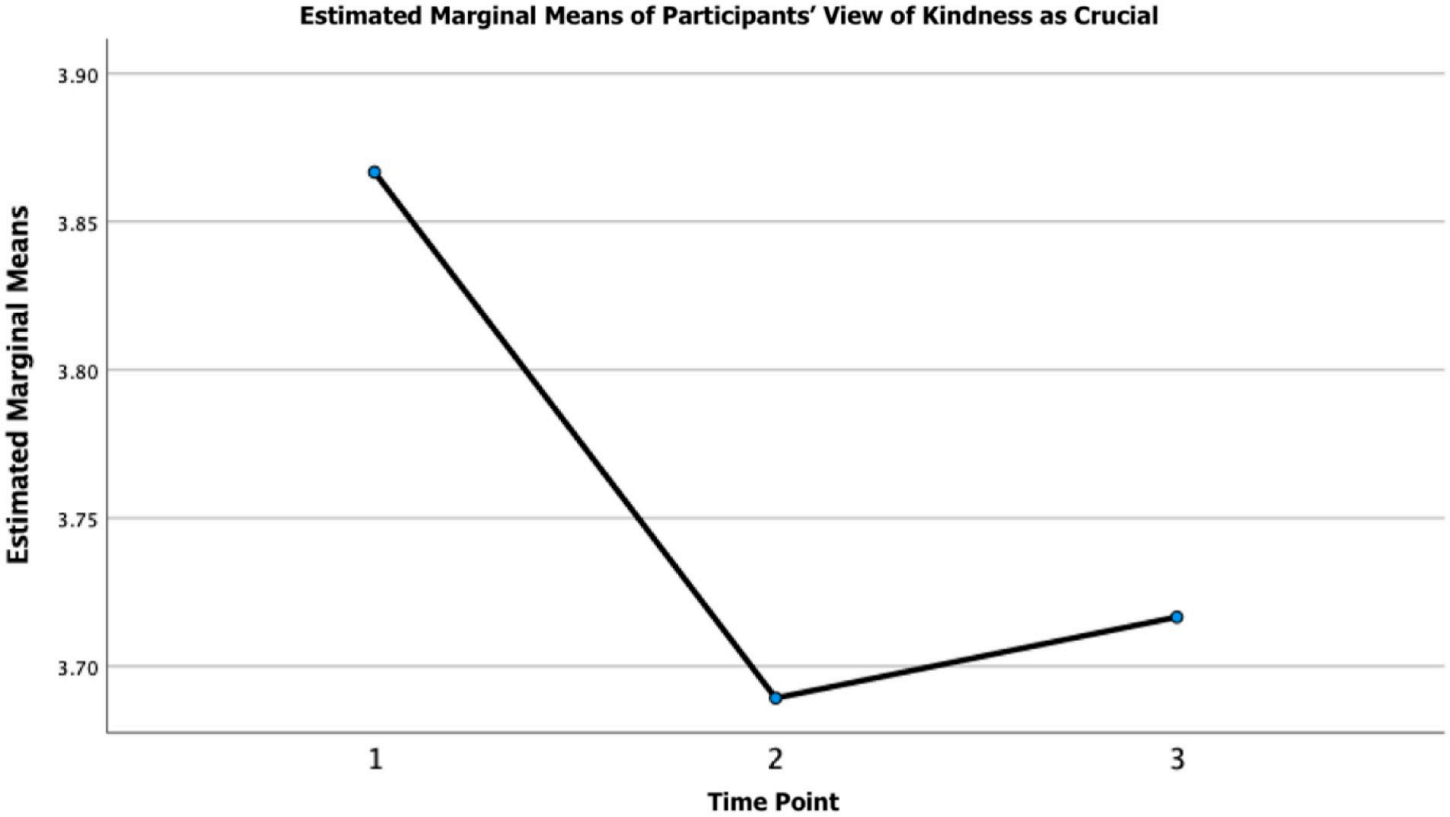
Participants’ view of kindness as crucial over time. *Note*. Time refers to the three time points (i.e., 1 = time point 1 [April 24-July 13, 2020]; 2 = time point 2 [July 29-August 30, 2020]; and 3 = time point 3 [July 29-August 30, 2021].

In addition to the above, a one-way repeated measures ANOVA revealed that there was a statistically significant difference in participants’ awareness of kindness over time (see Fig 2), participants’ self-reported engagement in deliberate acts of kindness (see Fig 3), and participants’ views of kindness as crucial over time (see Fig 4). Post hoc testing revealed that participants’ awareness of kindness decreased significantly from T1-T2 (M_difference_ = 0.27, *p* = < 0.001, 95% CI = 0.23 to 0.31) and from T1-T3 (M_difference_ = 0.22, *p* = < 0.001, 95% CI = 0.17 to 0.27) and increased from T2-T3 (M_difference_ = -0.05, *p* = 0.012, 95% CI = -0.10 to -0.01). Moreover, post hoc testing revealed that participants’ engagement in acts of kindness decreased significantly from T1-T2 (M_difference_ = 0.10, *p* = < 0.001, 95% CI = 0.05 to 0.15) and from T1-T3 (M_difference_ = 0.11, *p* = < 0.001, 95% CI = 0.05 to 0.16). Post-hoc testing revealed that participants’ views of kindness as crucial decreased significantly from T1-T2 (M_difference_ = 0.18, *p* = < 0.001, 95% CI = 0.12 to 0.23) and from T1-T3 (M_difference_ = 0.15, *p* = < 0.001, 95% CI = 0.09 to 0.21). The mean, standard deviation, and the *F*-ratio of the one-way repeated measures ANOVA for the global kindness questions can be found in Table 3.

### Qualitative

Four themes and eight subthemes emerged from the data: (1) examples of prosocial behaviour; (2) kindness from various perspectives (subthemes: receiving kindness, giving kindness, witnessing kindness); (3) shift in prosocial behaviour over the pandemic (subthemes: initial shock of COVID-19, kindness as a global phenomenon, rise of individualism, small-scale acts of kindness, no change in kindness); and (4) prosocial burnout.

#### Examples of prosocial behaviour

**Fig 5:**
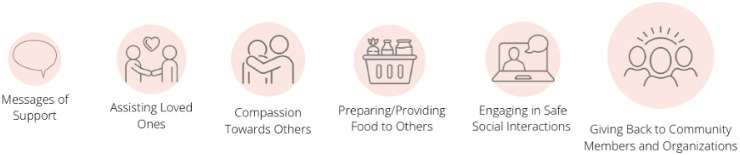
Examples of participants’ prosocial behaviour described during focus groups (n = 42).

Participants described various examples of prosocial behaviours engaged in throughout the COVID-19 pandemic. Summative content analysis revealed the following examples: messages of support (e.g., encouraging signs in windows; *n* = 15), assisting loved ones (e.g., babysitting for family members; *n* = 21), compassion towards others (e.g., tolerance of differing views; *n* = 22), preparing or providing food to others (e.g., grocery shopping for other households; *n* = 23), and giving back to community members and organizations (e.g., ‘free stuff’ Facebook groups; *n* = 45). The size of each icon in Fig 5 represents the number of times the corresponding example of prosocial behaviour was mentioned by participants, in relation to one another.

#### Kindness from various perspectives

Participants described different forms of kindness, including the experience of being on the receiving end of a kind act, the person engaging in the kind act, as well as the impact of witnessing kindness. Regarding the impact of being the kindness receiver, one participant (N23) reflected on how grateful they were for family support, and how much they missed it since the death of a generous loved one. They provided the following example:

…my mom passed away in August, not COVID-related, but totally unexpected. Um, and she used to watch my kids, you know, two or three days a week, and my, my mother-in-law, stepped in and said, ‘I’m going to be here on these, these days so you can work, and you can do whatever you need to do’. And I just remember feeling such an overwhelming, you know, gratitude for that. And I just, I couldn’t believe the amount of emotion that I was feeling.

Another participant reflected on how they are more aware of receiving kindness since the start of the pandemic as they routinely yearned for human connection; this participant noted, “I feel like I’m craving that connection or, you know? … So, when I do receive something, I am very aware that that’s super special because I am in need” (N33).” While relatively few participants described the impact of receiving kind acts, the majority of participants emphasized the benefits of being the person engaging in acts of kindness. One participant provided the example of donating clothes, saying, “I was able to donate a whole bunch of clothes to an organization… It was for people who needed clothes if they were in a fire or homeless… And it felt good to give” (N12). Another participant described how volunteering their time provided them with a sense of “purpose” when they did not have employment (N11). A few participants engaged in kindness as a coping mechanism and way to de-stress. This was emphasized by one participant (N34) who said:

I found… the best way to address my COVID despair was doing COVID-related acts of kindness… I did all the grocery shopping for my elderly grandma because it wasn’t safe for her…that’s kind of my takeaway—COVID-related acts of kindness were like, the best way to deal with my COVID despair and using my privilege in ways that I could help a little bit.

Similarly, another participant described how kindness was a stress reliever, saying, “Everybody that you do these little things [kindness] for are very appreciative and in the long run it’s also really good for me because it really helps me to destress” (N36). One individual (N39) decided to shift their focus to what they *could* do during the pandemic, which helped them cope with stressful pandemic experiences:

So, what I found I had to do is just focus on what I could do, like to make other people happy. So, a lot of that was just little things like, like, you know, dropping off little surprises to my dad on the doorstep, or you know, to an elderly aunt… And I would send …little surprise gifts to family out of town, like all the little kids and that kind of thing… Like to get away from all that really stressful negative feelings that was going on within the family. And I really just had to put the focus on, you know, doing good things for other people, and that really helped me to get through all of that.

Another participant highlighted the mutual benefit for both the giver and receiver of kindness, noting, “I realize, you know, in order to make you feel better sometimes its making other people feel better too… And then you get that effect where, you know, we build each other up” (N40). Interestingly, many participants reported experiencing benefits when witnessing acts of kindness without actually engaging in them. One participant (N8) described how seeing/hearing about acts of kindness provided them with hope:

It kind of gives you hope. You see all this negative news out there and then when you see, or you hear about those acts of kindness, it just kind reminds you that it’s not all negative news that, there, there is still good out there.

Another participant echoed this sentiment, explaining how “seeing people doing good things, seeing people be kind to others, considerate to others, doing their best just to do everything they can” helped to combat the negativity associated with the pandemic (N38). One participant highlighted that “seeing people being very kind and very good to each other and supporting local businesses and doing everything they can to make other people’s lives better [has been] super pivotal in keeping [them] positive through [the pandemic]” (N37). A few participants emphasized the value of community connectedness experienced when people are kind to one another. One individual stated that “we need each other [and] we need community in these kinds of times” (N4) while another participant (N9) expressed how kindness has brought people together:

My observation is that we … tend to live in a very me-centered world. You know, and these acts of kindness, and people gathering in really unique ways and checking on their neighbours and all of these things that we’ve seen on social media and the news firsthand. It’s, it’s so affirming and heartwarming and wonderful to see that people do still care. And, you know it took a pandemic for us to see that, you know, there’s still a lot of love in the world.

To summarize the sentiments of participants regarding the profound impact of engaging in acts of kindness, one participant stated, “kindness is always something that when you give it out, you get it back ten times more” (N41).

#### Shift in prosocial behaviour over the pandemic

**Fig 6:**
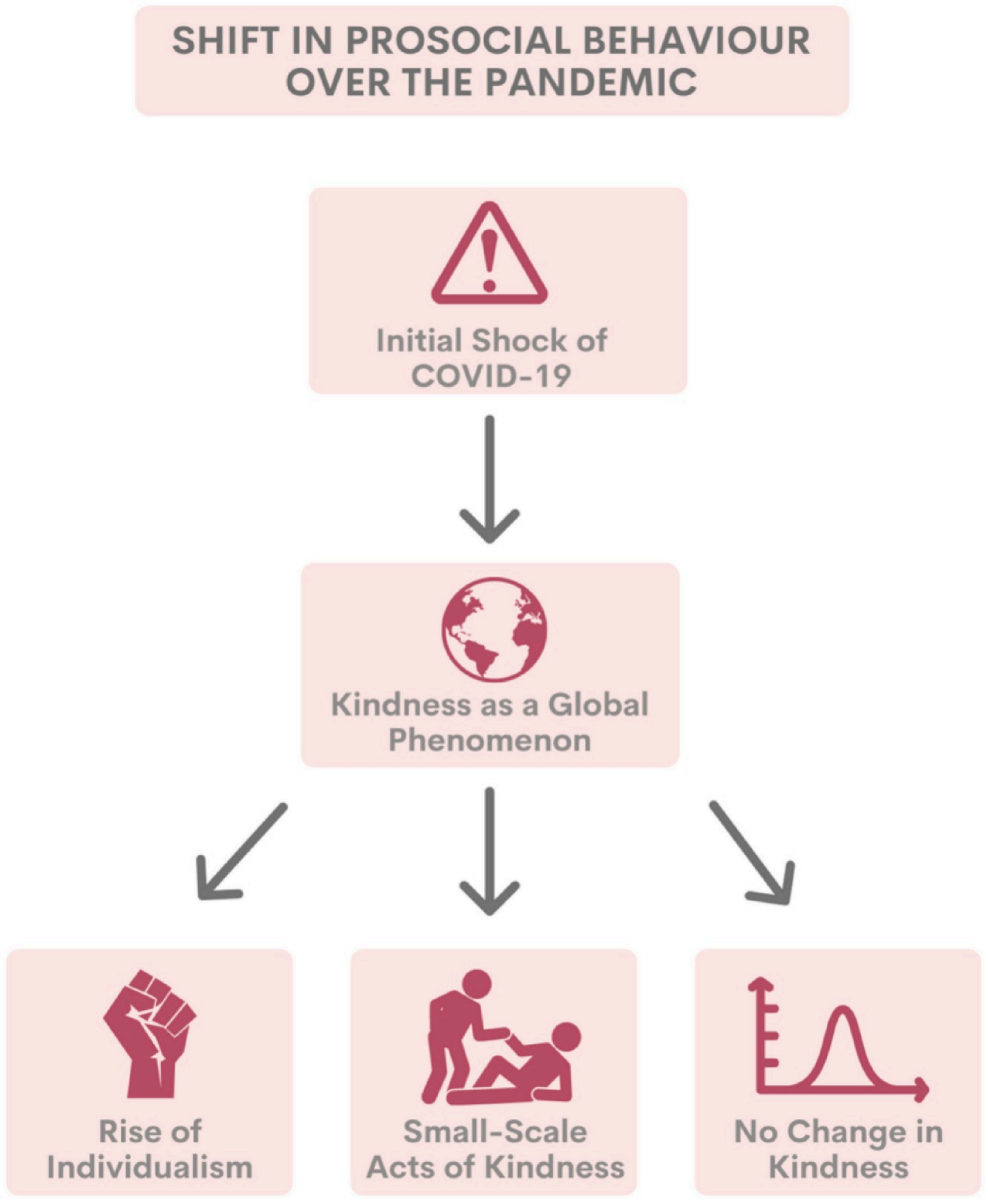
Flow diagram depicting shift in prosocial behaviour over the pandemic.

Participants across all focus groups discussed how prosocial behaviour (inclusive of acts of kindness) looked different as the pandemic continued. This shift in prosocial behaviour is depicted in the flow diagram found in Fig 6. Participants highlighted that initially there was a shock associated with COVID-19, which impeded their ability to engage in prosocial acts. This was emphasized by one participant (N24) who said:

…we witnessed hoarding at the beginning of the pandemic which made me supremely uncomfortable, and made me wonder if there was something more that I could be doing. I think the other thing is, I mean at the very beginning of the pandemic, it felt paralyzing, like you couldn’t even do anything. So, when you say that… the study found that people felt they could do more or be more as it [the pandemic] went on [regarding prosocial behaviour increasing over time], that doesn’t surprise me. Given how, you know, paralyzed in fear… our experience was at the beginning. As things began to open up, we could do more, we could pick up more supplies, we could at least drop something off on a doorstep…

Another participant (N34) explained how they perceived prosocial behaviour to evolve following the initial shock of the pandemic: “So at first, everybody sort of hunkered down, and then once they kind of got their bearings everybody wanted to know what they could do so there was a lot of prosocial behaviour” (N30). Following the initial shock of the COVID-19 pandemic, participants identified a rise in prosocial behaviour and described this as a “we’re all in this together” mentality. Some referenced prosocial behaviour as a collective phenomenon as emphasized by one participant (N5) who said:

I think what I’ve noticed as the shift is, at the beginning it was very global in the prosocial behaviour. So, I live very close to [city hospital], and I remember being slightly annoyed [at the beginning of the pandemic], but also really happy, with the week-long parade days that we had. There was a week where one day it was the ambulances that did the parade by the hospital, and through the hospital, and the university campus, and then by my house… And then the next day it was fire trucks, and the next day it was the city dump trucks, and the next day it was just other big trucks. And… I was like, ‘oh, my, how many groups of people are going to be driving down my house?’ And also how incredible is the support being shown for the healthcare workers who are putting themselves in danger every single shift, right?

This sentiment was shared by another participant who reflected that, “in the beginning we needed each other, as like a global community” (N18). Similarly, one participant said, “I feel like in the beginning we were a really unified group, you know? Back in the 14 days to flatten the curve we were all, ‘Rah! Rah! We’re in this together!’” (N27). The rise in prosocial behaviour and acts of kindness during the early stages of the pandemic was a shared sentiment as another participant (N21) noted:

And then we did have some families in our neighbourhood who did get hit with COVID, and there was a lot of sharing, there was a lot of support, it’s like [modulates tone], ‘oh do you need anything, let me drop it off at your doorstep, you know, and I’ll text you and then I’ll run away so that we don’t actually have to see each other and I’ll make sure that it’s all clean and sanitized’… and I just felt like in our little neighbourhood anyways, there was a lot of coming together, and a lot of trying to figure out how do we help each other through this.

While participants described how kindness started as a global phenomenon, many emphasized how prosocial behaviour changed over the course of the pandemic. For some, they recognized the rise of individualism and subsequent decrease in kindness, while others acknowledged that prosocial behaviour was still apparent, but had shifted to be more personal/small-scale. A small number of participants felt as though prosocial behaviour did not change, but rather people got used to witnessing and engaging in it. One participant (N26) noted that the “prosocial behaviours were a lot more at first [at the beginning of the pandemic]”, and another participant (N34) echoed this comment, saying:

…at the beginning you saw a lot more kindness [compared to now], whether it was directly by witnessing it among the people you know, or… stuff you see that goes viral online or stuff in the news… acts of kindness were really prominent and they were really part of the COVID narrative at the beginning.

Similarly, another participant (N25) noted how kindness shifted from a global phenomenon to one of individualism:

It just feels like, yeah, everybody was really united at the beginning and getting through this and then, you know, you would see lots of it in the media, or I did at least, and really now I don’t see any of that, maybe I’m just missing it, maybe I’m watching the wrong things, I don’t know but all I’m seeing is, you know, convoys and this huge division of, you know, [sigh] people calling other people anti-maskers and people throwing out all of these judgments towards other people. It’s like you’re either on this side, or you’re on this side, with like a few people in the middle… Like I don’t see this ‘togetherness’ that we saw at the beginning… I can’t even remember the last time I saw, you know, people coming together.

One participant described how kindness is no longer at the forefront, noting, “I just feel like overall, it’s kind of fallen off the radar a bit. And I’ve been seeing a fair bit of unkindness in social media, particularly in my day-to-day interactions with people” (N37). The unkindness experienced by this participant was not uncommon, as another individual noted that “it feels as though a lot of people are in it for themselves” (N25). Another participant (N21) reflected on the change in media headlines, saying:

I think at the beginning, there was a lot more mass media attention to how we were banding together and being kind to each other. And then as the pandemic went on and, you know, like it was just, it was just sad, and it just got really hard to take, so there might have been more kindness, but it was getting kind of quashed and hidden under everything else.

The feeling of kindness being overshadowed by negativity was shared among many, as another participant (N34) reflected on how kindness was hindered with the change in public health measures:

Once people stop wanting to do public health measures, which essentially to me communicates like, I don’t care about other people, it’s harder. Even if the quantity of prosocial behaviour hasn’t changed it’s harder to see it, because it is almost like it’s outweighed by the exponential increase of, it feels like, the anti-vax, anti-science, anti-COVID group.

Despite some participants witnessing a decline in prosocial behaviour or feeling as though kindness was no longer at the forefront of their pandemic experience, several participants acknowledged that kindness shifted from a global phenomenon to small-scale acts. Specifically, one participant (N37) noted that rather than prosocial behaviour being overshadowed by unkindness, they perceived kindness to take a new form:

I think that there’s other types of kindness happening for sure… People are supporting local businesses, and a lot of people are trying their best to, you know, wear their masks and get vaccinated, and encourage their friends and family to do likewise which I appreciate. But I’d say overall there’s less of that initial like ‘we’re in this together’ feeling.

Another participant (N5) suggested that prosocial behaviour became more personal and small-scale over time, saying:

What’s happening now is prosocial [has become a] personal behaviour… The tolerance for people who are different than you, the little rocks in your neighbourhood, keeping the magic alive for all these holidays for kids, which is wonderful… So the prosocial behaviour, I think, has gone from a really big picture down to personal interactions.

The experience of prosocial behaviour shifting from more of a “big picture” phenomenon to personal interactions was a commonality among participants. Namely, one participant noted that “it [prosocial behaviour] just has kind of shifted maybe a little bit from, so much of a community support to kind of just supporting individuals” (N27). Another participant (N11) recognized that while they were seeing kindness less in the news, they were more aware of the small-scale acts saying:

…we see less in the news but then, if you look around you like I think there’s more awareness of the little acts of kindness that people are doing… I think that it shifted from a global vision to a neighbourly vision.

These smaller acts of kindness described by participants took many forms. Specifically, one participant (N5) emphasized the genuine nature of small acts of kindness saying:

So now they [acts of kindness] are no longer big and grand with the parades, and the blue ribbons around the trees, but they are small, and they are pointed, and they are genuine, which I think is important from a perspective of everybody who’s involved… Whether it’s tolerating your family members who feel differently than you but you’re still having them over for dinner because you love them or it’s cutting those folks free from you because it’s easier for everybody to not have the tension of that relationship.

On the topic of this apparent shift in prosocial behaviour from more global to more individual, many participants viewed tolerance towards others with different viewpoints as a personal act of kindness. This shift was described by one participant (N4) who emphasized:

At the beginning it was more of those overt actions. Looking around how I could give, even with my church community… But I’m learning in all of this how to be more patient with other people. [I’m] less likely to maybe get upset that something isn’t going my way, or a line’s too long, or like whatever the things that were that would typically just kind of just get on my nerves. It’s not to say that they don’t get on my nerves now, they still do, but there’s kind of this piece at the forefront of everything that everyone’s kind of suffering right now. And everyone is kind of trying to persevere through this and aiming for this light at the end of the tunnel whatever it’s going to be.

Having tolerance for others was viewed as an individual form of kindness by another participant who highlighted that the pandemic has given them more perspective in that sense. This participant (N8) noted:

I’m just more cognizant of if someone is not kind [compared to the beginning of the pandemic], or if I see a lack of kindness, I maybe understand or take a step back and say ‘You know what? Maybe they’re just having a bad day, or maybe this is affecting them in such a negative way that I just have to be a bit more understanding of that’. Whereas I think before the pandemic if someone would have been unkind, I just would have thought, ‘Oh what a jerk!” and I would just move on… Whereas I think, for me anyway, it’s [the pandemic] given me a bit more perspective that way.

While most participants described how kindness shifted over the pandemic, some individuals suggested that there was no change in kindness. One participant suggested that “the sense of decrease [in kindness] is just that we’re not seeing this level of ‘newness’ that we saw at the beginning of the pandemic” (N4). Similarly, another participant highlighted that while it may feel as though prosocial behaviour has declined, perhaps “we are comparing it to what it could have been or used to be” (N21). The feeling of kindness being a ‘new normal’ was common as another participant (N38) noted:

I’m not sure it means that they’ve [acts of kindness] actually really slowed down… I think a lot of it just became sort of normal. People sort of found their support groups and maybe there just wasn’t as much a need to… for lack of a better word, advertised that that’s what was happening, right? It just kind of became ‘Okay, we’re in this groove now and here we go’.

This same participant described how kindness became “routine” such that “we maybe now do it without even really thinking too hard about it” (N38).

#### Prosocial burnout

Some participants experienced what was referred to by N15 as prosocial burnout over time. Specifically, prosocial burnout might help to explain some participants’ perceptions about prosocial behaviour reducing over the course of the pandemic. This was underscored by one participant who said, “I think that at the beginning of the pandemic people had a lot more capacity to hold space for other people because they were scared but not exhausted at that point” (N25). Several participants expressed feeling fatigued, with one individual (N24) summarizing sentiments from their focus group as follows:

…at the beginning [of the focus group] you asked how we were, and we said we were burnt out, and we were tired, and we were numb. I don’t know how easy it is for me in that state to recognize kindness or pull out the moments in my day where I experienced kindness, because I’m just so frazzled and tired.

Another participant (N10) described feelings of exhaustion due to the length of pandemic and the toll it continues to have on those in the medical field:

I think just people are exhausted. I think they’re starting to get drained. I have a lot of family members that are in the medical field, and they are just burnt out, you know? They appreciated all these acts of kindness in the beginning, but it’s just three years in now, it’s a lot for them.

Similarly, one individual felt as though kindness had “receded” due to “exhaustion from being two years into the pandemic and some of the other challenging negative storylines” that were in the news (N35). Another participant described being in “survival mode”, such that while there may have been kindness around them, they had a hard time “seeing the bigger picture” (N23). Lastly, one participant (N15) described the toll that engaging in kind acts had on them:

I think that part of it is that… the fatigue is so relevant now that it’s almost like we still have to do these things, not that we shouldn’t be kind to each other all the time, but the fact that we’re still in this crisis pandemic and having to think outside the box to have these prosocial interactions, is a bit fatiguing itself. The fact that this has been going on for two years, that, whereas these prosocial acts are not less valid now, but they just seem a little less impactful.

## Discussion

The purpose of this mixed methods research was two-fold: (1) to quantitatively assess adults’ prosocial behaviour over time during the first 16 months of the pandemic in Ontario, Canada; and, (2) to more deeply explore, via focus groups, a sub-sample of Ontario adults’ lived experiences of prosocial behaviour. The majority of participants included in this study identified as female, which should be considered in the context of study findings. Quantitatively, participants’ prosocial behaviour increased significantly from April 2020 to August 2021; however, participants’ awareness of kindness, engagement in deliberate acts of kindness, and view of kindness as crucial during the pandemic decreased significantly over time. Qualitatively, participants also described a shift in prosocial behaviour throughout the pandemic, although some felt the shift was based on perception versus reality.

While many participants expressed in the focus groups that they witnessed less engagement in deliberate acts of kindness over time, their overall PSA scores indicate that participants’ engagement in prosocial behaviour increased from April 2020 to August 2021. This finding aligns with work conducted by Vieria and colleagues [47] who conducted a study with 600 adults in the United States and found that those who perceived COVID-19 to be a threat were more likely to engage in everyday altruism. It is thus possible that participants’ self-reported increase in prosocial behaviour over time can be explained by the rise in COVID-19 cases during the data collection period, such that participants in the current study felt threatened by the pandemic and engaged in prosocial behaviour to combat this feeling. It is worth noting that prosocial behaviour and participants’ experiences of it might have aligned with the stressors experienced during different waves of the pandemic, and their emotional responses to COVID-19 case count fluctuations [39,41]. Some participants expressed how kindness shifted over the pandemic, suggesting that perhaps it did not decline; rather, acts of kindness were smaller or less apparent because individuals got used to them. While there are no longitudinal studies to date that report on prosocial behaviour over the pandemic, Tekin and colleagues [48] compiled altruistic stories during COVID-19 from individuals in various countries (i.e., India, Australia, United States, and England). After conducting a qualitative content analysis of 104 altruistic stories, the authors found that community members and volunteers engaged in prosocial behaviour during the COVID-19 pandemic, with material resources representing a common type of support [48]. This finding aligns with the current study, as participants self-described as volunteers and offered support to other community members. Similarly, participants in the current study offered material resources by donating clothes, providing public health supplies (e.g., masks) to others, and buying groceries for individuals who were unable to do so. Participants also described support that was shown for frontline workers and provided examples such as seeing signs in front of houses for healthcare heroes and parades for frontline workers outside of hospitals. This finding aligns with Tekin and colleagues’ [48] study findings as the authors noted that frontline workers were among the groups that received the most support during the pandemic globally. The authors suggested that there was an increase in community-based support during the pandemic, which took the form of individual volunteers aiding community members [48]. Similarly, in the current study participants described kindness as a global phenomenon and expressed a collective “we’re all in this together” mentality at the beginning of the pandemic, pointing towards community-connectedness and supporting one another during the early stages. The quantitative findings revealed that prosocial behaviour increased over the course of the first year of the pandemic, while qualitatively participants felt as though it had shifted/decreased by the second year. The qualitative findings might help to explain the quantitative increase in prosocial behaviour, as participants described a global prosocial movement of sorts at the beginning of the pandemic, which transitioned to less grand and more individualized gestures. Although some participants reported feeling as though prosocial behaviour decreased over time, other individuals noted that perhaps people became too exhausted to recognize prosocial behaviour in the same ways they did at the start of the pandemic. This finding is not surprising given that Haktanir and colleagues [49] explored pandemic fatigue among adults (*N* = 516) and found a significant correlation between pandemic fatigue and intolerance of uncertainty, fear of coronavirus, and self-care. Moreover, engaging in prosocial behaviour can decrease one’s stress levels and feelings of depression while increasing optimism, especially when the act of kindness is done out of concern for the wellbeing of others [50]. Therefore, it is not surprising that when reflecting on their engagement in acts of kindness, some participants in the study described prosocial behaviour as a stress reliever and/or coping mechanism during the pandemic.

During the focus groups, participants described various acts of kindness engaged in over the course of the COVID-19 pandemic. Many participants noted ways that they gave back to their communities and local organizations, with most describing online communities as one method of support (e.g., donating resources in ‘free stuff’ Facebook groups). Though less frequent, participants also described acts that involved physical contact with other people, including offering childcare to family members and preparing food for others. These findings align with the work conducted by Aresi and colleagues [51], who explored adults’ (*N* = 2,562) patterns of prosocial behaviours during collective quarantine conditions in Italy. The researchers found four classes of prosocial behaviour: (1) money donors; (2) online and offline helpers; (3) online health information sharers; and, (4) neighbour helpers [51]. Of the four classes, offering help to others both online and in-person was reported most frequently [51]. This finding aligns with the current study as giving back to community members and organizations was reported most often, which included online interactions and support of others.

As noted above, some participants described a rise in individualism over the course of the pandemic, as well as prosocial burnout due to exhaustion. Specifically, participants noted a shift in what was being presented in the media, recognizing that at the beginning of the pandemic what was being reported in the media was more positive; however, over time, participants noted that they were seeing an increase in ‘unkindness’ and divisiveness portrayed in the media. Many participants described that their consumption of negative news impacted how they viewed kindness and prosocial behaviour. This finding is not surprising and corresponds with work conducted by Buchanan and colleagues [52], who investigated the emotional consequences of exposure to COVID-19-related news among adults in England. The authors conducted two studies (*N*_study 1_ = 402; *N*_study 2_ = 813) and participants in each study were assigned to one of three groups: (1) COVID-19 information; (2) COVID-19 kindness; or, (3) no information (control; [52]). The authors found that consumption of COVID-19-related news resulted in immediate and significant reductions in optimism and positive affect, when compared to the no information exposure group [52]. Further, they found that exposure to COVID-19-related acts of kindness did not elicit the same negative consequences [52]. Thus, it may be the case that participants in the current study felt as though the shift in media consumption towards more COVID-negative news impacted their views of kindness and contributed to prosocial burnout/exhaustion. Furthermore, it is possible that the perceived rise in individualism and prosocial burnout was due to ongoing stress associated with the COVID-19 pandemic [53]. It is possible that during the earlier stages of the pandemic (2020–2021; when the quantitative data was collected) participants were distressed coupled with high case counts, contributing to prosocial burnout. Although case counts were no longer at the forefront of the media at the time that qualitative data were collected (March 2022), it is possible that being two years into the pandemic created exhaustion for many and a de-sensitization of sorts.

### Limitations

This study is not without limitations. First, the researchers created the three global kindness questions that were asked, and therefore, the three questions were not validated. Moreover, *honesty demands* (per Bates [37]) were employed, however, the self-reported data collected in this study still lends itself to social desirability bias. When interpreting study findings, it is also worth noting that the quantitative data were collected at a different timeframe than the qualitative data (April 2020-August 2021 vs. March 2022, respectively). While the focus groups served to supplement quantitative findings by capturing participants’ lived experiences of prosocial behaviour throughout the pandemic, participants spoke to their prosocial behaviour at the different time points (i.e., when the survey data was collected versus when the focus group data was collected). This served as a limitation as the COVID-19 context differed across time points, which could impact the interpretation of study findings. Furthermore, because the quantitative findings informed the focus group guide, it is possible that participants’ responses were biased, given that they were told the survey data prior to sharing their personal experiences. While this was done intentionally, future studies may wish to separate quantitative and qualitative data.

## Conclusion

The prosocial behaviour of participants, who were mostly female-identifying, increased from April 2020 to August 2021. Further, participants described a shift in their prosocial behaviour over the course of the pandemic, from kindness being a global phenomenon to more small-scale acts of kindness, with some participants noting a rise in individualism and prosocial burnout contributing to a decline in prosocial behaviours. Participants also recognized kindness from various perspectives and reflected on the impacts of receiving, giving, and witnessing acts of kindness. This is the first study to provide insight into the long-term effects of the pandemic on adults’ prosocial behaviour and should be leveraged to help understand how individuals, and more specifically those who identify as female, respond in times of crises.

## Supporting information

S1 Appendix (DOCX)
