# Supplementary material for: ‘When you give kindness out, you get it back ten times more’: Ontario adults’ prosocial behaviour during the first 16 months of the COVID-19 pandemic
Source: PLoS One. 2023 Jul 17;18(7):e0288720. doi: 10.1371/journal.pone.0288720 (PMC10351708; doi:10.1371/journal.pone.0288720)
Supplement: S1 Appendix — (DOCX) [file pone.0288720.s001.docx]

**Appendix A**

**Focus Group Questions**

1. Although the specific focus of today’s discussion is prosocial behaviour, we’d like to start a bit more general. We are now at about two years since COVID-19 started, and we’d like to start with a few minutes of no-pressure chatting about how everyone is doing in general?
2. This question is a two-part question: Earlier in this study we asked a series of questions pertaining to your prosocial behaviour. Data analysis revealed that participants’ prosocial behaviour **increased** over time. (1) To what extent does this feel accurate or inaccurate regarding your own personal experiences of engaging in prosocial behaviour over the course of the pandemic? (2) What reasons do you think this might be the case (for you and/or for others)?
   1. What would you like to add to this?
   2. Please say more about…
   3. Please elaborate…
3. What role do you think prosocial behaviour, including acts of kindness, has played in your own personal experience of the pandemic (either by you and/or by others)? What’s been/is important about it to you personally?
   1. In what ways have you seen prosocial behaviour?
      1. How did witnessing prosocial behaviour influence you?
   2. What’s an example of prosocial behaviour that you experienced?
   3. In what ways has prosocial behaviour influenced your overall wellbeing during the COVID-19 pandemic?
4. This question is a two-part question: Earlier in this study you were asked the extent to which you were aware of kindness around you during the COVID-19 pandemic. Data analysis revealed that participants’ awareness of kindness around them **decreased** over time. (1) To what extent does this feel accurate or inaccurate regarding your own personal awareness of kindness over the course of the pandemic? (2) What reasons do you think this might be the case (for you and/or for others)?
   1. What would you like to add to this?
   2. Please say more about…
   3. Please elaborate…
5. This question is a two-part question: Earlier in this study you were asked the extent to which you viewed kindness as a crucial component of your COVID-19 pandemic experience. Data analysis revealed that participants’ view of kindness as a crucial component of their pandemic experience **decreased** over time. (1) To what extent does this feel accurate or inaccurate regarding your own personal view of kindness as crucial over the course of the pandemic? (2) What reasons do you think this might be the case (for you and/or for others)?
   1. What would you like to add to this?
   2. Please say more about…
   3. Please elaborate…
6. What else haven’t I asked you that I should have?

**General Probes:**

- What’s an example of…?
- Please say more about…
- Please elaborate…
